# Social isolation and suicide risk: Literature review and perspectives

**DOI:** 10.1192/j.eurpsy.2022.2320

**Published:** 2022-10-11

**Authors:** Chloé Motillon-Toudic, Michel Walter, Monique Séguin, Jean-Daniel Carrier, Sofian Berrouiguet, Christophe Lemey

**Affiliations:** 1 Mental Health Department, Brest Medical University Hospital, Brest, France; 2 Department of Psychology, Université du Québec en Outaouais, Gatineau, Québec, Canada; 3 Department of Psychiatry, Faculty of Medicine and Health Sciences, Université de Sherbrooke, Sherbrooke, Québec, Canada; 4 LaTIM, INSERM, UMR 1101, Brest, France

**Keywords:** Social isolation, social support, suicide attempt, suicide ideation, suicide risk

## Abstract

**Background:**

Suicide is a major public health problem and a cause of premature mortality. With a view to prevention, a great deal of research has been devoted to the determinants of suicide, focusing mostly on individual risk factors, particularly depression. In addition to causes intrinsic to the individual, the social environment has also been widely studied, particularly social isolation. This paper examines the social dimension of suicide etiology through a review of the literature on the relationship between suicide and social isolation.

**Methods:**

Medline searches via PubMed and PsycINFO were conducted. The keywords were “suicid*” AND “isolation.”

**Results:**

Of the 2,684 articles initially retrieved, 46 were included in the review.

**Conclusions:**

Supported by proven theoretical foundations, mainly those developed by E. Durkheim and T. Joiner, a large majority of the articles included endorse the idea of a causal relationship between social isolation and suicide, and conversely, a protective effect of social support against suicide. Moreover, the association between suicide and social isolation is subject to variations related to age, gender, psychopathology, and specific circumstances. The social etiology of suicide has implications for intervention and future research.

## Introduction

According to the World Health Organization (WHO), suicide is defined as the act of deliberately ending one’s life [1]. Epidemiologically, suicide is a leading cause of premature death that would be preventable [2]. Every year, 800,000 people die by suicide worldwide, corresponding to one death every 40 s [1]. For every suicidal death, an estimated 20 suicide attempts take place [1]. All regions of the world, developed and developing countries alike, are affected by this phenomenon [1]. Moreover, suicide significantly impacts both the individual’s close circle of friends and family and society as a whole [1]. This epidemiological data makes suicide a critical public health problem on a global scale. One of the objectives stated in WHO’s Mental Health Action Plan 2013–2020 was to reduce suicide rates by 10% by 2020 [2].

Suicide is associated with clearly established clinical risk factors, a history of suicide attempts being the most consistent among them [1]. A meta-analysis has shown that among suicide-risk factor associations, the strongest is the one between suicide and previous suicide attempts (odds ratio [OR] = 16.33; 95% confidence interval [CI] = 7.51–35.52) [3]. Another significant association reported in said study [3] is between suicide and mood disorders, including depressive disorders (OR = 13.42; 95% CI = 8.05–22.37).

In addition to clinical risk factors, the social environment, including interpersonal relationships, correlates to suicide and can be effectively targeted from a prevention perspective [1]. This literature review focuses specifically on social isolation as a potential suicide risk factor.

E. Durkheim was the first to emphasize the importance of social variables in the etiology of suicide in his 1897 sociological study [4]. In 2005, T. Joiner [5] put forward a new theoretical model of suicide, the interpersonal theory of suicide. Their studies concur in highlighting the significant role that social isolation plays in the suicidal process.

In light of the international scope of the literature on the link between suicide and social isolation, this study examines this relationship by drawing on published research regardless of the nature of the sources. The objective is to identify specific characteristics mentioned in the international literature regarding the relationship between suicide and social isolation. Theoretical bases on the subject will be addressed, and perspectives on intervention, especially prevention, and future research will be discussed.

## Method

We conducted a literature review concerning the association between a social variable, that is, social isolation, and suicide. We followed the Preferred Reporting Items for Systematic Reviews and Meta-Analyses (PRISMA) guidelines [6] to ensure methodology appropriateness and accurate results reporting.

We conducted searches in two databases: Medline via PubMed and PsycINFO. During an informal preparatory exploration of the literature, the keyword “isolation” emerged as the term that was typically used to refer to the concept under study, leading us to adopt the search formula: “suicid*” AND “isolation.” Articles published through April 2020 were considered in the final search conducted in May 2020. We did not limit the database search by publication date.

The eligibility criteria used to select the articles included were the following:

(a) Articles that, according to their abstract, dealt mainly with the relationship between the social variable, that is, social isolation or other related social concepts, and any of the stages of the suicidal process (suicide ideation, suicide attempt, completed suicide) or more generally with suicide risk; (b) Articles published in English, for international visibility; and (c) Articles whose abstracts were available in either database.

We exported article publication data (authors, title, journal, year of publication) from the searches into a word processing program (Word 2016). We removed duplicates during data processing after reading the title and abstract of each article.

Whenever there was doubt about whether an article should be included, a discussion was held between two independent experts until they reached a consensus.

## Results

**Figure 1.**
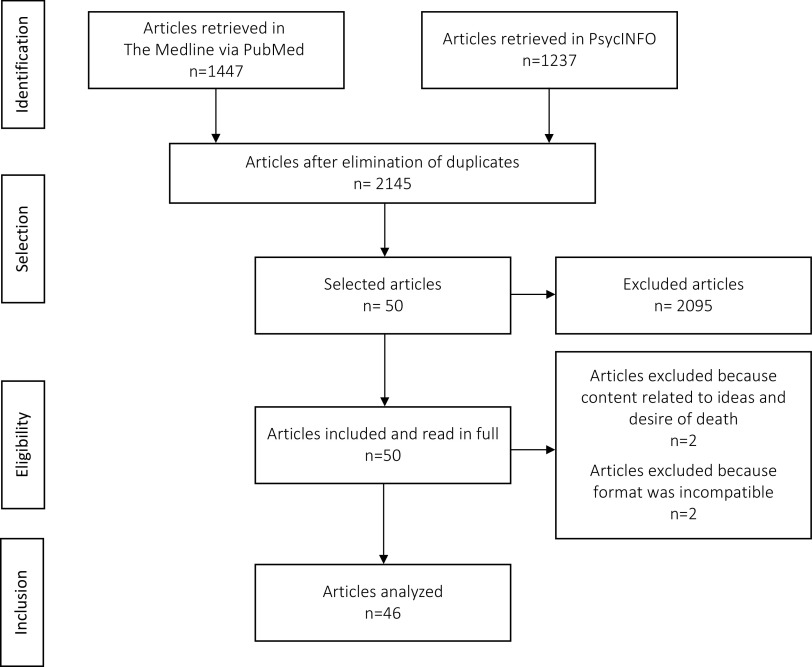
Flow chart.

A flow chart of the research process is presented in Figure 1.

Out of the 2,684 articles whose abstracts were retrieved, 50 were read in full, and 46 were included for analysis. We excluded two articles because the topics were the desire for and ideas about death rather than ideation relating to actively ending one’s own life. In fact, those studies had excluded participants with suicidal ideation. We excluded two other articles based on their publication format: a letter and an editorial.

Methodological data for the analyzed articles are reported in Table 1.

**Table 1.** Methodological data of the articles analyzed.

Article	Main objective	Design	Suicidality measures	Social isolation measures	Main results
Acosta et al. [7]	Examining the effect of ethnicity on thwarted belongingness, perceived burdensomeness, and current suicidal desire. Hypothesis: Being part of the Hispanic community has a moderating effect on thwarted belongingness and perceived burdensomeness. In contrast, individuals with high levels of thwarted belongingness and perceived burdensomeness exhibit higher levels of suicidal desire.	Cross-sectional study comparing students of different ethnicity, that is, students from the Hispanic community and students who are part of the non-Hispanic Caucasian community. (Self-administered questionnaires).	Suicidal desire assessment based on the Depressive Symptom Inventory-Suicidality Subscale (DSI-SS).	Interpersonal factor assessment (thwarted belongingness and perceived burdensomeness) through the Interpersonal Needs Questionnaire (INQ).	The Hispanic community had significantly lower levels of suicidal desire and perceived burdensomeness than the non-Hispanic Caucasian community. The levels of thwarted belongingness were not significantly different between the two groups. The non-Hispanic Caucasian community with high levels of thwarted belongingness and perceived burdensomeness also had higher levels of suicidal desire. Thus, ethnicity did moderate the effect of interpersonal factors on suicidal desire, but not in the way that was expected, with an opposite direction from the initial hypothesis.
Arango et al. [8]	Examination of the relationship between bullying and social connectedness, and suicide ideation and attempts.	Cross-sectional study. The following variables were controlled for: gender, age, ethnicity, and perception of public assistance.	The Columbia Suicide-Severity Rating Scale: Screen Version (adapted CSSRS) defined three groups at risk for suicide: a group with no recent suicidal ideation or history of suicide attempts (*N* = 202), a group experiencing only recent suicidal ideation (71), and a group with a history of suicide attempts with or without recent suicidal ideation (48).	Social connectedness was assessed by self-reporting based on the UCLA Loneliness Scale-Revised.	The analyses revealed that high levels of harassment and low social connectedness were significantly associated with suicide ideation and suicide attempts. Social connectedness did not appear to be a moderator in the relationship between harassment and suicide risk. The results showed a continuum in the severity of involvement in harassment and the social connection associated with suicide risk.
Armstrong et al. [9]	Examining risk factors for suicidality, i.e., geographic isolation and distance from home to school, and protective factors, i.e., engagement in meaningful extracurricular activities and social support.	Cross-sectional study. (Self-assessment questionnaires).	Suicidal ideation assessed through the Suicidal Ideation Questionnaire (SIQ).	The Centre of Excellence for Youth Engagement (CEYE) measured youth engagement. The Social Support Questionnaire (SSQ) measure of low social support represented social isolation. Participants were also asked about the geographic location where they lived and how far their home was from school.	The prevalence rate of critical suicidal ideation was 8.3% with a sex ratio of 1.86:1 (female:male). Distance from home to school emerged as a significant risk factor for suicidal ideation, only for males. Engagement in structured extracurricular activities was significantly associated with less suicidal ideation. Distance from home to school indicated significantly lower activity engagement. Engagement in meaningful extracurricular activities may mediate the relationship between distance from school and suicidal ideation. Neither geographic isolation nor social support reliably predicted suicidal ideation in the male sex.
Bille-Braheet al. [10]	Analysis of the level of social integration of suicidal individuals compared to the general population.	Cross-sectional analyses.	Psychiatric admission following a suicide attempt.	Three relational domains explored: the immediate environment of family, friends and neighbors, the work environment, and the community.	The results showed that suicidal individuals have inadequate contact in all three domains with a larger gap in the degree of community integration between suicidal individuals and the general population. In addition, the total level of social integration significantly lower among suicidal individuals compared to the general population suggests that suicidal individuals do not compensate for poor integration in one domain by a stronger integration in another domain.
Bock [11]	Significance of various types of group membership for suicide in older adults.	Cross-sectional, case-control study. Comparison between 188 suicide and 2,544 non-suicide seniors. Field survey.	Suicides on death certificates and funeral home director records.	Several types of social relationships studied: marriage, kinship networks, and organizational membership.	Each type of social relationship was independently and significantly associated to suicide with membership in organizations that played a key role in this study than the other two types of social relationships. The suicide rate increased from 16 to 114 depending on whether or not they belonged to membership or non-membership in organizations, respectively. The highest suicide rate (62) was in the lower levels of social classes in which there was less chance of being married, to have family in the community or to belong to community organizations.
Bornheimer et al. [12]	Exploring the relationships between positive symptomatology (hallucinations and delusions), depression, social isolation and suicidal ideation.	Cross-sectional study. Covariates included in the study were ethnicity, gender, and age.	Suicidal ideation measured by an item assessing whether the person has ever seriously thought about suicide.	Social isolation assessed through the following three items: “I keep to myself even when others are around,” “I feel awkward in social situations” and “I prefer activities that I can do by myself.”.	Approximately 11, 18.9, and 0.2% of participants had suicidal ideation, depression, and a diagnosis of schizophrenia and/or psychotic disorder, respectively. Results showed direct and significant relationships between positive symptomatology, depression, and suicidal ideation and indirect relationships mediated by social isolation. Hallucinations, delusions and depression were significantly associated with the three items characterizing social isolation, and these three items were significantly associated with suicidal ideation. Social isolation thus played a partial mediating role. In terms of total effects, depression mediated by social isolation increased the risk of suicidal ideation the most, compared with positive symptomatology.
Bränström et al. [13]	Analysis of the disparity in suicide ideation and attempts between Lesbian/ Gays/Bisexuals and heterosexuals and analyzing barriers to societal integration as potential explanatory factors for this disparity, beyond the contribution of already established risk factors. Risk factors also studied: psychological (depressive symptoms and substance abuse) and interpersonal (exposure to discrimination, victimization or threat of aggression and lack of social support).	Cross-sectional study. Data for the study were obtained from the cross-sectional Swedish National Public Health Survey. (National registries and self-assessments).	Suicidality examined by two measures: suicidal ideation and suicide attempts in the past 12 months.	Barriers to societal integration refer to the degree to which a person assimilates societal norms and expectations and are defined by four variables: single or not living with a partner, not living with children, lack of trust in society and unemployment. Lack of social support was assessed using two questions, “Do you have someone you can share your innermost feelings with and confide in?” and “Can you get help from one or several persons if you have practical problems or are ill?”.	By logistic regressions and in comparison, with heterosexuals, gays and lesbians on the one hand and bisexuals on the other had significantly more suicide ideation and attempts. Among LGB people, all psychological, interpersonal and societal integration risk factors, except unemployment for gays and lesbians, were significantly more frequent than among heterosexuals. These analyses were adjusted by covariates: age, gender, education level, individual income, being urban, and country of birth. Examining mediation by multiple mediation analyses and after controlling for covariates including education level, individual income, and being urban, the results showed that psychological, interpersonal, and barrier to societal integration factors mediated the association between sexual orientation and suicidality. Thus, barriers to societal integration emerged as explanatory factors for the disparity in suicide ideation and attempts between LGB and heterosexuals, even when controlling for recognized mediators.
Calati et al. [14]	Overview of the link between social isolation and suicidal thoughts/behaviors.	Narrative review of the literature: systematic reviews, meta-analyses, narrative reviews, original observational studies with large samples (*N* ≥ 500), and some qualitative studies.	All forms of suicidal outcomes.	Any form of social isolation or loneliness.	Main social constructs associated with suicidal outcomes: marital status, living alone, social isolation, loneliness, alienation, and belongingness. Strong association between the objective condition and the subjective feeling (loneliness) of being alone, and suicidal outcomes, in particular suicide ideation and suicide attempts. Loneliness, studied in most studies, showed a major impact on suicide ideation and suicide attempts.
Chen [15]	Eleven studies in which ostracism is experimentally manipulated using several methods in an attempt to establish a cause-and-effect relationship between thwarted belongingness and suicidal thoughts, which are in turn assessed using various measures.	Experimental study. Random assignment to study conditions.	Assessment of suicidal thoughts using various measures: Implicit Association Test of Suicide, self-reports, hypothetical stress situations.	Ostracism manipulation through several methods: recalling a past experience, imagining a future experience, and inducing a sense of being ostracized at the time of the study. In addition, use of the Ostracism Experience Scale to assess feelings of being ostracized.	The results showed that “without social ties, life lacks meaning, thus activating suicidal thoughts.” It has been shown that ostracism increased suicidal thoughts and that this relationship was mediated by the perceived meaning of life. In order to check the robustness of the results, alternative hypotheses were tested: if ostracism induced a change in the perception of the acceptability of suicide, if ostracism increased suicidal thoughts due to negative mood, whether the perceived meaning of life did not explain the effect of ostracism on suicidal thoughts after control of depressive affect, if the perceived meaning of life did not explain the effect of ostracism on suicidal thoughts after control of the four basic needs and K. Williams’ mood. These four hypotheses were rejected in three of the studies. The assertiveness exercises reduced the effects of ostracism on suicidal thoughts.
De Grove [16]	Investigating the relative contributions of anomie, isolation, and psychopathology to suicide rates.	Cross-sectional study.	Suicide rate = average annual death rate per 100,000 population from deaths recognized as suicides (E950–E959, Eight Revision International Classification of Diseases).	Isolation indicator = percentage of “heads of household” living alone or with “non-relatives” only in their household.	After inclusion in the model of the high median age of the population as an exogenous variable for suicide, this variable was found to have the largest effect on suicide rates with both a direct and indirect causal effect. The indirect causal effect of age on the suicide rate consists of two positive contingents contributed by isolation and psychopathology and a negative contingent contributed by anomie. After considering age, anomie, isolation and psychopathology were revealed, in this order, as causal factors for suicide. Concerning more specifically isolation, although affected by age, it contributed independently and significantly to suicide.
Duberstein [17]	Examining the relationship between suicide and social integration.	Case-control study. Comparison between suicide victims and living controls. Psychological autopsy method.	Deaths by suicide determined by medical examiners.	Social integration defined by family and social/community indicators.	After statistical adjustment for the effects of affective disorder and employment status, marital status, social interaction, and religious involvement were associated with suicide. The addition of substance abuse disorder as an adjustment variable removed religious involvement from the association with suicide.
Fisher [18]	Exploration of various correlates of the sense of belonging.	Cross-sectional study. (Structured clinical interviews and self-assessments). Covariates controlled were marital status, social support, life stress, depression, and hopelessness.	The Beck Scale for Suicide Ideation (BSSI) measuring suicidal ideation.	The Sense of Belonging Instrument-Psychological Experience (SOBI-P) for the assessment of the sense of belonging. The Multidimensional Scale of Perceived Social Support for a person’s perceived level of support from family, friends, and others. The Modified Life Experience Scale for current and life stressors, including relationship problems. In addition, participants were interviewed to collect demographic and social network information, including the presence or absence of confidants in their lives.	The results of the hierarchical multiple regression analysis indicated that sense of belonging did not predict current suicidal ideation or history of suicide attempts beyond the other covariates. Sense of belonging was significantly and directly associated with depression and hopelessness, and indirectly with suicidal ideation. Depression was significantly related to hopelessness. Depression and hopelessness were significantly associated with suicidal ideation. The presence or absence of a confidant was significantly associated with a sense of belonging and depression. The relationship between the sense of belonging and suicidal ideation was mediated here by depression and hopelessness.
Fullen [19]	Examining the relationship between thwarted belongingness, perceived burdensomeness and psychological distress, and suicidal risk in homebound older adults.	Cross-sectional study. Data collection for a large longitudinal study on the effectiveness of suicide prevention strategies targeting older adults. (Self-assessments).	Suicidal risk measured using the Suicidal Behaviors Questionnaire—Revised (SBQ-R). Suicidal risk threshold: score ≥ 7.	Interpersonal Needs Questionnaire (INQ) assessed thwarted belongingness and perceived burdensomeness. Risk thresholds: score of 17+ on the Perceived Burdensomeness subscale or 36+ on the Thwarted Belongingness subscale.	The results showed that 15.62% of the elderly had scores above the suicide risk threshold, 23.73% reported a history of suicidal ideation or behavior, 13.18% revealed the possibility of future suicide attempts with 2.43% characterizing their future suicide attempt as “probable” or “very probable.” 20.30% of the older adults met the risk threshold on at least one subscale of the INQ with thwarted belongingness scores of 8.20–63. Among the study subjects, thwarted belongingness, perceived burdensomeness, and acquired suicidal ability were prevalent. Older adults with scores above the suicide risk threshold reported significantly higher levels of thwarted belongingness and perceived burdensomeness. No differences regarding gender and ethnicity were found for suicidal risk, thwarted belongingness, and perceived burdensomeness.
Hedley [20]	Examining the risk of loneliness and the protective potential of social support in depression and suicidal ideation in individuals with autism spectrum disorder. Autistic traits were also considered.	Cross-sectional study in which participants were recruited among those participating in two Australian longitudinal studies of autism.	The Patient Health Questionnaire (PHQ) to assess depression and suicidal ideation.	The University of California Los Angeles Loneliness Scale-Short Form (ULS-8) for assessing feelings of loneliness and social isolation, and the Social Support Questionnaire-Shortened Version (SSQ-6) for assessing social support with the number of social supports and satisfaction in relation to social support.	Nearly 49% of participants were above the clinical threshold for depression and nearly 36% had recently presented suicidal ideation. Regression analyses showed that loneliness, satisfaction with social support, and autism spectrum disorder traits were significantly associated with depression. After adding depression and autism spectrum disorder traits to the model for suicidal ideation, the significant relationship between satisfaction with social support and suicidal ideation had disappeared and only depression was significantly associated with suicidal ideation. Trajectory analysis revealed that: autism spectrum disorder traits were related to depression, even after controlling for loneliness and satisfaction with social support; the influence of number of social supports on depression was mediated by loneliness and satisfaction with social support; and, the influences of loneliness and satisfaction with social support on suicidal ideation were mediated by depression. In addition, autism spectrum disorder traits predicted less satisfaction with social support and more loneliness. Only depression predicted suicidal ideation. Thus, this study highlighted that loneliness and social support were protective and risk factors, respectively, for depression in adolescents and adults with autism spectrum disorder and that depression mediated the relationship between these social variables and suicidal ideation. Participants who reported being in a relationship did not differ on the main study variables study variables than those who were not in a relationship.
Hunt et al. [21]	Testing the interpersonal theory of suicide via the Interpersonal Needs Questionnaire (INQ).	Cross-sectional study. (Self-assessment).	Suicide Ideation Questionnaire-Junior > 31.	The Interpersonal Needs Questionnaire (INQ).	This study identified a third factor playing a role in adolescent suicidality, perceived isolation, derived from items 11 and 12 of the INQ, originally proposed as part of the thwarted belongingness. The results indicated that perceived burdensomeness and the interaction between perceived burdensomeness and perceived isolation predicted suicidal ideation, while controlling for depression and demographic factors, which was not the case for thwarted belongingness or perceived isolation.
Jacobs and Teicher [22]	Study of suicidal people’s evolution process through comparing the life histories of adolescents having received hospital care following a suicide attempt and control adolescents.	A case history approach to the study of adolescent suicide attempts. (Accounts of teenagers and parents).	Suicide attempts.	Social relationships. The authors focused on the sequential order of particular events rather than on an independent event at the root of the suicidal behavior.	The investigation identified the three stages of a common process to suicidal individuals that led them to a progressive isolation from significant social relationships contributing to the suicide attempt: a long history of problems, followed by an escalation of problems, in excess of those usually associated with adolescence and, finally, by chain reaction, a dissolution of all remaining significant social ties in the days and weeks before the suicide attempt. The suicide is the result of a rational decision-making process in which the desire to die is not the motivation but death is imposed in front of the progressive failure of all attempts to adapt to the primary problem of existence which is the maintenance of social relationships.
Joiner et al. [23]	*Study 1* tests the assumption that the joint presence of a low level of belonging and the perception of burdening others are predictors of suicidal ideation. *Study 2* tests the assumption that the simultaneous presence of a low sense of belonging and perceived burdensomeness will not result in a suicide attempt unless the individual has developed the capability to engage in suicidal behavior.	Two cross-sectional studies.	*Study 1:* Current (past month) suicidal ideation. *Study 2:* Current suicide attempts.	*Study 1:* Measurement of family social support using the modified and shortened version of the Provisions for Social Relations Scale. *Study 2:* Measurement of thwarted belongingness and perceived burdensomeness using the Suicide Probability Scale (SPS).	The first study argues that the interaction, via indirect measures, of low belongingness and perceived burdensomeness predicts current suicidal ideation in a large and representative sample of the population from which it is drawn, beyond measures of depression and unmoderated by gender and ethnicity. The second study demonstrates that the 3-way interaction of low belongingness, perceived burdensomness, and history of suicide attempts predicts current suicide attempts in a sample of young adults with a recent suicide attempt or severe suicidal ideation, over and above measures of depression and other variables implicated in suicidality.
King and Merchant [24]	Exploration of social and interpersonal influences on suicidality. Three lines of analysis: parent, family, and peer relationships, physical and sexual abuse, and peer victimization and harassment.	Review of the literature.	Suicidal ideation, suicide attempts, and deaths by suicide.	Social isolation was part of the “parent, family and peer relationships” focus.	The results of these studies highlight the importance of social variables, such as social integration, perceived family and peer support, childhood abuse and neglect, and peer victimization, in suicidality of adolescents.
Krauss and Krauss[25]	Examination of the thwarting-disorientation theory of suicide.	Literature review. Cross-cultural survey.	Suicide case histories.	Thwarting-disorientation situations.	Suicides are associated here with “thwarting-disorientation” situations.
Kwon et al. [26]	Analysis of the effects of formal and informal social relationships on suicidal ideation in older adults.	Cross-sectional study.	If the elderly person reported having thought about suicide since turning 60, they were considered to have suicidal ideation. (Self-evaluation).	Formal and informal relationships seniors have. Formal social relationships are ties to society through formal relationships with organizations or participation in social activities. Informal relationships are represented by ties to family, friends, and neighbors.	15% of the sample had suicidal ideation. Controlling for sociodemographic variables, informal social relationships with children, friends and neighbors were significantly associated with suicidal ideation, whereas informal social relationships with siblings and other relatives and formal social relationships were not. As for sociodemographic variables, older age, being male, rating one’s own health as poor, living in the city, and depression were significantly associated with the presence of suicidal ideation, whereas education, income, and employment status were not significant variables.
Logan et al. [27]	Examination of the relationship between suicidal ideation and various forms of social connectedness.	Cross-sectional study. Data were drawn from the Youth Violence Survey: Linkages Among Different Forms of Violence. Associations analyzed by logistic regression with control for demographics (age, gender, ethnicity), mental distress, substance use, and violence victimization. (Self-administered questionnaire).	Suicidal ideation assessed by whether or not a suicide attempt was seriously considered in the 12 months prior to the survey.	Social variables studied: friendships with offenders, parenting style (caring and/or monitoring), degree of social support, school connectedness, and number of perceived close friends.	17% of the population had seriously considered suicide in the past year. After controlling for all factors, suicidal ideation was negatively associated with school attachment and all parenting styles.
Masuda et al. [28]	Examining the relationship between online social networks and suicidal ideation.	Cross-sectional study. Data obtained from a leading social network service in Japan, Mixi.	A user belonging to at least one community included as suicidal is defined as having suicidal ideation. *N* = 9 990.	Independent variables: number of communities, degree (number of friends, a small degree is an indicator of social isolation), local clustering coefficient (transitivity or density of triangles around a user, a small value is an indicator of social isolation), homophily (proportion of friends with suicidal thoughts).	The results revealed that three variables contributed most to suicidal ideation, and in this order: increasing the number of communities to which a user belongs, intransitivity, and increasing the fraction of suicidal friends in the social network. The variables demographic of age and gender, and the number of friends had little effect on suicidal ideation.
Milner et al. [29]	Comparison of the number of social ties in the lives of people who have attempted to commit suicide or have died by suicide (cases) and living controls.	Population-based case–control study. Cross-sectional observational study. Adjusted for employment status, marital status, socioeconomic status, and diagnosis of an affective or anxiety disorder. (Psychosocial Autopsies).	Suicide cases determined by “intentional self-harm,” which is the terminology for suicide deaths in the National Coronial Information System (NCIS) database and in the coroner’s records. Cases of attempted suicides determined by admission to a hospital emergency department for medical care following intentional self-harm.	Primary exposure variable = social ties at the individual, community, and societal levels (number of ties a person had in their life). Self-assessments.	After adjusting for variables, those with 3–4 social ties were 74% less likely to commit or attempt suicide and those with 5–6 social ties were 89% less likely to commit or attempt suicide compared to those with 0–2 social ties. When the number of social ties was considered a continuous variable, the odds ratio was 0.39 per social tie. Compared to those with 0–2 social ties. When the number of social ties was considered a continuous variable, the OR was 0.39 per social tie. Observation of a dose-response relationship between the number of social ties and the risk of suicidal behavior.
Näher et al. [30]	Examination of potential associations between individual indicators of socioeconomic status and social isolation, and suicide rates. Initial hypotheses: lower levels of socioeconomic status, higher levels of social isolation, and their interactions are associated with higher suicide rates.	Cross-sectional study.	Suicide rates. Suicides defined by ICD 9 (E950–E959) in 1997 and by ICD 10 (X60–X84) as of 1998. Number of suicides: 149,033.	Social isolation characterized by being single, living in a single-person household, and having moved in the past year.	Positive effects of socioeconomic status and social isolation on suicide rates are demonstrated. However, the initial hypotheses are only partially verified. Regarding social isolation, the results reveal that for a 1% increase in one-person households, the suicide rate is increased by 1.65%, while for a 1% increase in the number of people who moved in the last year, the suicide rate is decreased by −2.13%.
Neuringer [31]	Examination of social isolation in three groups of individuals.	Projective test, the Make-A-Picture Story Test (MAPS).	Individuals having attempted to commit suicide.	Social isolation was defined in this study by the number of figures used and their social withdrawal attributes.	The results showed that the suicidal individuals used more figures than the other groups and that the social withdrawal attributes of these figures did not differ from those of the control patients. The social isolation of the suicidal patients was thus manifested, via the test by an increased use of adapted figures for interpersonal relations.
Sainsbury P. [32]	Examination of the hypothesis that “where social mobility and social isolation are pronounced, community life will be unstable, without order or purpose, and that this will be reflected to a greater or less degree in the suicide rates.”.	Study conducted in two parts. Part 1: Statistical correlation of suicide rates in the 28 London boroughs and the city with selected indices of their social characteristics. Part 2: Analysis of the antecedents of 409 suicides in North London.	Suicide rate.	Social mobility and social isolation.	The relationship between suicide and a solitary lifestyle, or social isolation, was “probably a cause and effect relationship”.
Ojagbemi et al. [33]	Determining the prevalence of suicide ideation, plans, attempts, and their predictors.	Cross-sectional study. Data from the Ibadan Study of Aging, a large community-based longitudinal study of the mental and physical health of people aged 65 and older.	The third version of the World Health Organization (WHO) Composite International Diagnostic Interview (CIDI) used to measure suicide ideation, plans and attempts of individuals aged 65 and older.	The Composite International Diagnostic Interview was used to assess the social network. Participants were required to indicate the frequency of contact with non-household family members and friends (less than once every 6 months vs. more than once every 6 months).	Suicidal ideation was common in the elderly: 4.0% with suicidal ideation, and 0.7 and 0.2% with plans and attempts, respectively. Being separated from one’s spouse, mainly by divorce or death, significantly predicted suicide planning and living in a rural area significantly predicted suicidal ideation. There was a linear relationship between residence and risk of suicidal ideation. The circumstances of social isolation and exclusion were therefore correlated with suicidal behavior in the elderly people.
Oliffe [34]	Exploring the various dimensions of the lived experience of social isolation involved in male suicidality.	Photovoice, a qualitative research method based on photographic data. Participants are invited to take photographs and then participate in an interview, to illustrate their experiences of suicidality and to enhance understanding of the relationship between male gender and suicidality.	X.	Examples and experiences of social isolation. “How did this isolation contribute to your suicidality?”.	Social isolation represented as composed of multiple dimensions, attached to each other, emanating from family, school and work, self-management, health care, idealized identity, and society. Social isolation is a powerful risk factor for suicide in humans and whose phenomenological understanding of it is essential to the examination of male suicidality.
Oliffe et al. [35]	Improved understanding of the link between masculinity and suicidality.	Photovoice, qualitative research method based on photographic data. Participants are invited to take photographs and then participate in an interview to illustrate their experiences of suicidality and thus enhance understanding of the relationship between male gender and suicidality.	X.	X.	Three interconnected themes characterizing male suicidality emerged from the analysis: injury, interiority and isolation. Isolation described corresponded to separation from others and was related to abandonment issues and lack of a job and/or partner. “Self-isolation” was explained as a protective strategy to relieve others and/or limit further exposure to harmful stimuli.
Poudel-Tandukar et al. [36]	Investigating the relationship between social support and death by suicide.	Japanese prospective study. Population-based cohort study. Controlled by numerous individual- and lifestyle-related factors.	Deaths by suicide identified on death certificates by International Classification of Diseases, 10th Revision (ICD-10) codes X60–X84.	Social support measured through a combination of four items resulting from self-esteem support, support from a confidant, and social isolation indices. Social isolation determined by not having a friend that the participant knew well enough to meet at least once a week and assessed by the item, “how many friends do you meet at least once a week?”.	A total of 180 suicides were recorded during an average of 12 years of follow-up. In the multivariate-adjusted models, men and women who had the highest level of social support had, respectively, 44 and 62% less likely to die by suicide, compared to those with the lowest level of social support. By type of social support, the following results were obtained: support from a confidant was not significantly associated with suicide in both sexes, esteem support was significantly associated with a lower risk of suicide in women but not for men, and having 4 or more friends was significantly associated with a lower risk of suicide risk of suicide for men but not for women, however a similar trend was still observed among women.
Purcell et al. [37]	Assessment of associations between lifestyles (“living alone” versus “living with other people”), family ties, and suicidal ideation.	Cross-sectional study. Age, sex, and marital status were controlled.	Assessment of suicidal ideation using the Scale for Suicide Ideation (SSI).	Assessment of family connectedness using the Reasons for Living Scale—Older Adult version (RFL-OA). Assessment of perceived social support based on the Duke Social Support Index (DSSI; Perceived Social Support (PSS) subscale).	Patients with strong family ties were significantly less likely to express suicidal ideation than those with weak family ties. This protective effect was greater for patients living with others than for those living alone. A significant interaction of lifestyle and family ties on suicidal ideation was thus demonstrated. In addition, high perceived social support was significantly associated with less expression of suicidal ideation but without a significant interaction of lifestyle and perceived support on suicidal ideation. Regardless of lifestyle, perceived social support was protective against suicidal ideation in this sample.
Roeder and Cole [38]	Simultaneous examination of thwarted belongingness, perceived burdensomeness, and hopelessness (assessed using the Beck Hopelessness Scale) as predictors of suicidal ideation.	Longitudinal study. Evaluation at two times, 4 months apart. (Self-assessment).	The Suicidal Ideation Questionnaire-Jr. (SIQ) to measure suicidal ideation.	Interpersonal Needs Questionnaire (INQ) for measuring thwarted belongingness and perceived burdensomeness.	When the three cognitive factors were examined separately, each predicted future suicidal ideation in both groups, whereas when the factors were examined together, none of the factors emerged as a single predictor. Thus, it was hypothesized and then demonstrated that a common underlying factor across all three cognitive variables significantly predicted suicidal ideation. Thwarted belongingness longitudinally predicted perceived burdensomeness and hopelessness. The results of this study led to support for a broader model in which thwarted belongingness, perceived burdensomeness, and hopelessness, factors from two different theoretical models, would interact together to form the basis of a superior negative belief system that would more accurately predict suicidal ideation.
Roma [39]	Exploration of suicide rates within Italian prisons with various detention conditions.	Cross-sectional study. Data from the Prison Department of the Italian Ministry of Justice on suicide and detention conditions.	Number of suicides.	Three main types of detention: without isolation in a large prison community, with short-term isolation, and in maximum-security solitary confinement.	The median suicide rates per 100,000 per year for the years 2004–2005 are 4.9 for the general population, 96.8 for non-segregated inmates, 232.8 for inmates in temporary segregation, and 353.4 for maximum security inmates.
Stanley [40]	Examining the relationship between suicide risk and firefighter status, wildland or non-wildland.	Cross-sectional study. Data from two large national surveys in the United States on firefighter mental health.	Suicidal risk assessed by the Suicidal Behaviors Questionnaire-Revised (SBQ-R).	Thwarted belongingness and perceived burdensomeness determined by the Interpersonal Needs Questionnaire.	Wildland firefighters had significantly higher mean levels of suicide risk compared with nonwildland firefighters with a mean total Suicidal Behaviors Questionnaire—Revised score of 7.40 for wildland firefighters and 5.78 for nonwildland firefighters, even after controlling for gender. Thwarted belongingness, but not perceived burdensomeness, appeared in the analysis as a potential mediator of the positive association between wildland firefighter status and increased suicide risk. Wildland firefighters had significantly higher mean levels of thwarted belongingness, while controlling for gender. Mediation analyses revealed that the direct effects of wildland firefighter status on suicide risk, controlling for thwarted belongingness, perceived burdensomeness, and gender, tended toward statistical significance; whereas the specific indirect effect of thwarted belongingness on suicide risk was statistically significant both in the model where thwarted belongingness and perceived burdensomeness were examined as parallel mediators and in the separate models where these two constructs were examined as individual mediators.
Tomek et al. [41]	Exploring the relationship between suicide ideation and attempts, and poverty and school connectedness.	Cross-sectional study. Data from the Mobile Youth and Poverty Study, a longitudinal study.	Suicidal ideation measured by the following self-assessment question: “In the past year, have you seriously thought about killing yourself?” Suicide attempt measured by the following self-assessment question: “Have you ever attempted suicide?”.	School connectedness measured by eight items adapted from Goodenow’s Psychological Sense of School Membership.	11% of the sample reported suicidal ideation and 9% reported a suicide attempt. Results showed that when school connectedness was higher, the odds of suicide ideation and attempts were significantly lower regardless of gender, with a consistent effect over time. Female gender was associated with significantly higher odds of suicide ideation and attempts compared with male gender.
Travis [42]	Which theory, E. Durkheim’s theory of social disorganization or M. Halbwachs’ theory of social isolation, best predicted suicides among a population in transition, the Alaska Natives?.	Cross-sectional surveys. (Social Autopsies).	Suicide stated as cause of death on death certificates.	Based on Halbwachs’ theory, suicides committed on vacation or living in a community settled in no longer than 5 years prior were related to social isolation.	The results tend to support Halbwachs’ theory as a better explanation of the overall suicide rate than Durkheim’s theory, but not significantly so. Both suicide percentages and suicide rates were higher for social isolation than for social disorganization from 1959 to 1984. Men committed suicide more than women, with an overall suicide rate of 74.5 per 100,000 for men and 19.6 for women. This study also identified the importance of the social dimension in male suicide by highlighting singlehood, unemployment, and culture as potential explanatory factors for this gender disparity in this population. A gender disparity in the specific rates of suicide related to social isolation was observed with 25.6 and 9.4 per 100,000 for men and women, respectively.
Trout [43]	Review of the literature on the role of social isolation in suicide.	Review of the literature.	Suicidal outcomes.	Subjective and objective measures of social isolation.	Social isolation is associated with suicide in a direct and fundamental way.
Tsai et al. [44]	Estimating the relationship between social integration and suicide mortality in women aged 18 and older (1992–2010).	From the Nurses’ Health Study, a national prospective cohort study carried out in the United States.	Suicide deaths as defined by the World Health Organization International Classification of Diseases, Eighth Revision (ICD-8), codes E950–E959.	Social integration measured using a seven-item index including marital status, size of social network, frequency of contact with social ties, participation in religious or other social groups.	The most socially isolated women represented the category in which the cumulative incidence of suicide was highest. After adjustment for several covariates, the result was the same with the lowest risk of suicide among participants in the highest category of social integration. Additional analysis, controlling for changes in the social integration trajectories of 65,507 participants and covariates, showed that those with the highest level of social integration also had a lower risk of suicide. Controlling for antidepressant medication and/or a history of physician-diagnosed depression did not alter the results.
Van Orden et al. [45]	Assessment of factors related to the interpersonal theory of suicide.	Case study (suicides)—controls (living). The database consists of psychological autopsies. Pre-existing data.	Suicides identified by medical examiners.	Three composite variables created for the purpose of analysis: thwarted belongingness, burdensomeness risk, and painful and triggering experiences.	On independent examination of all three variables, suicide victims had higher levels of all three variables compared to controls. When examined concurrently, only higher levels of perceived burdensomeness and painful and provocative experiences were associated with case status compared with controls. Neither thwarted belongingness nor depression were significant predictors.
Vanderhorst and McLaren [46]	Predicting suicidal ideation and depression based on marital status, social support resources, and sense of belonging.	Cross-sectional study. Gender, age, and education level were controlled.	Suicidal ideation was assessed through the suicide subscale of the General Health Questionnaire.	Sense of Belonging using the Sense of Belonging Instrument and social support resources using the social support subscale of the Coping Resources Inventory.	Results from the hierarchical multiple regression analysis indicated that only social support significantly predicted depression and suicidal ideation, such that having fewer social support resources was associated with higher levels of depression and suicidal ideation. Sense of belonging did not predict depression and suicidal ideation beyond social support.
Wyman et al. [47]	Analysis of the predictive value of structural characteristics of school networks for suicide ideation and attempt rates in schools.	Cross-sectional study.	Assessment of suicide ideation and attempts using the Youth Risk Behavior Survey measure.	Formation of social networks by participants nominating up to seven of their closest friends and up to seven trusted adults from among the people in the school. Social network measures: peer network integration, peer network centralization, peer network cohesion, suicidal student influence, student-adult networks, student-adult network centralization.	At the individual level, 8% of the participants reported having seriously considered suicide without actually committing suicide and 7% reported one or more suicide attempts. At the school level, the average rate of suicide attempts was 6.5% and the average rate of suicidal ideation was 8.5%. Weaker peer network integration and cohesion increased the likelihood of suicide ideation and attempts in both individual and school patterns. Survey results indicated that peer and youth-adult networks from the larger school scale influenced the rate of suicide attempts beyond individual student connections. After adjusting for depression and victimization by violence and harassment, estimates of the influence of network characteristics between youth and adults were little changed, while those for network variables between peers were reduced. The network characteristics associated with suicide attempts were in three domains: social integration in relation to thwarted relational needs, group cohesion and social influence of suicidal students.
Yong [48]	Determining the basic characteristics as well as the psychiatric factors associated with hikikomori.	Secondary analysis of a nationwide cross-sectional study. Data from the Survey of Young People’s Attitudes. (Self-administered questionnaires).	Items assessing suicide risk: “I often feel guilty towards family,” “I often feel that my life is suffocated,” “I wish to die,” “I always feel hopelessness,” and “I hurt myself.”.	Hikikomori defined in terms of the frequency of going out (“staying home most of the time”) and the duration of this behavior (“6 months or more”). Personal data retained and five different psychiatric factors studied including interpersonal difficulties. Items assessing interpersonal difficulties: “I am afraid of meeting others,” “I am anxious about the possibility of meeting people that I know,” “I am anxious about what others might think of me,” and “I cannot blend into groups.”.	Prevalence of hikikomori = 1.8% of which 41% have been in this state for more than 3 years. The multiple logistic regression analyses showed that after adjustment for age, gender, number of family members, social class, all psychiatric factors and history of psychiatric treatment, only interpersonal difficulties remained significantly associated with hikikomori. The addition of the history of psychiatric treatment as an adjustment variable removed the significant relationship between hikikomori and suicide risk. In terms of significance and strength of the associations, hikikomori is therefore associated mainly with interpersonal difficulties and then to suicide risks. Among the adjustment variables in the multiple logistic regression analyses, male gender and history of psychiatric treatment were significantly associated with hikikomori.
Yur’yev et al. [49]	Evaluation of the relationship between the economic (economic/employment) and social (social/welfare) dimensions of social exclusion and suicide mortality in Europe.	Analytical study.	Age- and sex-adjusted suicide rates for the last 5 years available for each country were obtained from the World Health Organization European Mortality Database.	E. “To what extent do you agree or disagree that social benefits and services in [country] prevent widespread poverty?” F. “To what extent do you agree or disagree that social benefits and services in [country] lead to a more equal society?” G. “To what extent do you agree or disagree that social benefits and services in [country] make it easier for people to combine work and family life?” These questions and social spending were considered as indicators of the “social/welfare” dimension of social exclusion.	The hypothesis that social exclusion is a risk factor for suicide mortality was broadly supported with suicide mortality rates negatively influenced by the economic and social dimensions of exclusion in both men and women. However, some relationships were not statistically significant: the relationship between the economic and social dimensions and the suicide mortality rate for women, and the relationship between employment rates and the economic/employment dimension in either the male or female model. The two dimensions of social exclusion had a significant and strong influence on male suicides, whereas these same dimensions had a nonsignificant and smaller magnitude influence on female suicides. Meanwhile, the social dimension had more weight and tended towards significance regarding female suicide, compared to the economic dimension. The remaining influence of GDP was positive in both models.
Zamora-Kapoor et al. [50]	Examination of the relative importance and mediation of social isolation, suicide exposure, and being overweight in suicidal ideation.	Retrospective population-based cohort study. Data from the first wave of the National Longitudinal Study of Adolescent to Adult Health.	Suicidal ideation as measured by the question, “At any time in the past 12 months, did you seriously think about trying to kill yourself?”.	Social isolation measured by the degree of agreement or disagreement with the statements, “I feel socially accepted” and “I feel like I belong in school.”.	Prevalence of suicidal ideation: 17.2% among Alaska Natives and 13.6 in the Caucasian population. In the final model, exposure to suicide by friends had the largest effect. Exposure to suicide of family members was still significantly associated with suicidal ideation. Social isolation was significantly and positively associated with an increase in the likelihood of having suicidal ideation with an OR = 2.03 for participants feeling socially unaccepted and an OR = 1.33 for participants who did not feel as part of the school. In this final model, being overweight also increased the risk of suicidal ideation. Trajectory analyses revealed that social isolation, exposure to suicide and overweight were significantly and directly associated, without mediation, to suicidal ideation among Native Americans in Alaska. Among Caucasian youth, social isolation and exposure to suicide were significantly and directly related to suicidal ideation. Suicide exposure and overweight were significantly associated with social isolation.
Zaroff et al. [51]	Assessment of interpersonal variables, perceived burdensomeness and relationship status, and other variables such as depression, hopelessness, gender, and region of residence prior to university enrolment in Macao, as risk factors for suicidality, after controlling for response bias via the Marlowe-Crowne Social Desirability Scale (MCSDS).	Cross-sectional study. (Self-assessment).	Beck Scale of Suicidal Ideation (BSSI) used as a measure of suicidality.	Relationship status: single or in a relationship. Perceived Burdensomeness Examination: Interpersonal Needs Questionnaire (INQ). The internal consistency of the thwarted belongingness items, also derived from the INQ, was below acceptable values and therefore the variable was not assessed as a risk factor.	Only perceived burdensomeness and relationship status significantly predicted suicidality. Neither depression nor hopelessness, nor gender, nor region of residence before college entry predicted suicidality.
Zhang and Jin [52]	Examining the relationship between three interpersonal factors and suicidal ideation.	Cross-sectional study. (Self-administered questionnaire).	Two items assessed suicidal ideation: one item asked the respondents how many times during the past year they had thought about killing themselves and the other how many times during the past year they had prepared or had planned to kill themselves.	Three interpersonal factors: difficulty interacting, interpersonal conflict and social isolation. Each factor measured by several items.	28.3% of the participants reported having thought about committing suicide at least once in the past year and 7.7% having prepared or planned a suicide. Differences for these two items were observed according to gender with a higher proportion of women responding positively. Interpersonal conflict had the strongest direct and total predictive effect on suicidal ideation, whereas social isolation was the weakest predictor among the three interpersonal factors. All interpersonal factors were significantly associated with the frequency of suicidal ideation, but the predictive value varied among the factors. Interpersonal conflict and difficulty interacting were both directly positively and indirectly related to suicidal ideation, unlike social isolation, which showed only an indirect effect, through depression and self-esteem. Participants with high levels of social isolation were more likely to have high risk of having high levels of depression and low self-esteem, with a positive correlation between the level of depression and suicidal ideation and a negative correlation between self-esteem and suicidal ideation. The total effect of interpersonal conflict was 0.570 on suicidal ideation, the total effect of interaction difficulty interaction was 0.303 and the total effect of indirect effect of social isolation was 0.040.

*Note*: X: information not available.

### Theoretical basis

Among the different visions concerning suicidology research, the one stemming from sociology and from E. Durkheim’s investigation on the influence of social causes on suicide is major [25]. By establishing that suicide rates are inversely correlated with social integration, E. Durkheim was the first to highlight the social dimension as a causal factor in suicide [4]. Durkheim described the type of suicide linked to a lack of social integration as egotistic [4]. Consequently, social isolation and social support seem to appear respectively as risk and protective factors for suicide. Durkheim’s hypothesis of social integration as a cause of suicide, based on a model of social disorganization, was re-examined by M. Halbwachs in his comprehensive statistical study of the causes of suicide [53]. Halbwachs proposed a psychosocial theory of suicide, arguing that a suicidal act should be considered from two different angles, one relating to individual causes and the other to social causes [43]. According to T. Joiner’s interpersonal theory of suicide, social connection is also a key element in the suicidal process. He posits that simultaneous thwarted belongingness, that is, a feeling of no longer being an integral part of a group, and perceived burdensomeness, that is, a feeling of being a burden to others, are at the root of the emergence of suicidal ideation. The concomitant presence of the cognitive factors of thwarted belongingness and perceived burdensomeness leads to the emergence of suicidal desire, which, with the acquisition of suicidal capability, often developed through repeated suicide attempts, may evolve into suicidal acting out. These theoretical models have been explored and largely validated by a number of studies. Therefore, they provide robust explanatory frameworks to explain the relationship between social isolation and suicide risk. Research on the relationship between either social isolation or social support and suicidality is abundant and spans several decades. From P. Sainsbury’s 1955 work [32] to a review of the literature dating from 1980 [43] to an experimental study conducted in 2020 [15], many researches draw the same conclusion and consider social isolation as a major suicide risk factor.

There is no consensus on the definition of social isolation, but it can be described as a state in which interpersonal contacts and relationships are disrupted or non-existent [43]. In the articles reviewed, social isolation and related concepts were assessed in various ways, ranging from qualitative descriptive variables such as social networks, single relationship status, and living alone, to quantitative assessment scales, the most widely used of which was the Interpersonal Needs Questionnaire (INQ) [7, 21, 38, 40, 51]. The INQ, a self-administered questionnaire derived from the Interpersonal Suicide Theory [54], includes 15 self-reported items measured on a 7-point Likert scale anchored with 1 (Not at all true for me) to 7 (Very true for me). The first six items of the INQ correspond to perceived burdensomeness and the remaining nine to thwarted belongingness.

Several factors that further characterize the relationship between suicide and social isolation emerged from the articles reviewed. These include age and gender, as well as psychopathology and specific circumstances.

### Role of age and gender

Suicidality affects individuals of any age and gender. However, depending on these demographic factors, there are differences in the relationship between suicidality and social isolation [1, 10]. For example, one study showed that age and gender impact social isolation [10], with evidence that levels of social integration varied according to these factors. Therefore, age and gender have a confounding potential that justifies their routine control in research on the relationship between suicide and social isolation [21, 23].

Two age groups require special attention: individuals aged 70 and older, who have the highest suicide rates, and younger individuals, aged 15–29, in whom suicide is the second leading cause of death. In addition to the high suicide rates in the elderly [1], suicide attempts in this population are more often fatal, with a ratio of suicide attempts to the suicide of 4:1 [19]. This reinforces the importance of the suicidality problem in the elderly. Social isolation seems to play a central role in suicidality for both seniors and adolescents, but the social contexts inherent to age are different. On the one hand, aging is inevitably accompanied by the loss of interpersonal relationships, the most impactful being the loss of a spouse. On the other hand, adolescence is a period of life marked by disruptions in social bonds, which can become weaker. Our review suggests that, in these two age categories, the family circle is a powerful vector of social support [11, 24, 26, 27, 37]. Informal relationships, especially with children, are even more protective of suicidal thoughts in older adults living alone than formal relationships created officially by society like paid caregivers [26]. Schooling has also been found to be a protective social factor for adolescents [9, 27, 41, 47]. Involvement in meaningful extracurricular activities [9] as well as school determinants of adolescent social networks, particularly with adults [47], should be leveraged in the development of interventions to prevent suicidality in young individuals. Therefore, seniors and youth are both age groups for which the fight against social isolation looks like a major lead for suicide prevention.

Social isolation affects both male and female suicidality, but the extent of its influence differs by gender [36, 49]. Our review reveals that social isolation and suicidality appear to be more strongly associated with men than women. This result should be accounted for when considering that men have higher rates of suicide mortality [1]. In high-income countries, three times more men die by suicide than women, and in middle- and low-income countries, the suicide rate for men is 1.6 times that of women. In male suicidality, social isolation seems to be a leitmotif of various life areas ranging from family to society [34, 35]. Therefore, men represent a population at higher risk of suicide partly because of social isolation, to which they are probably more exposed or vulnerable. These findings warrant promoting social bonding in suicide policy interventions with this subgroup.

### Psychopathology and specific circumstances

#### Psychopathology

Other factors, psychopathological in particular, may also be at play in the relationship between social isolation and suicidality. The scientific literature presents evidence of a bidirectional relationship between social isolation and mental health [30]. Indeed, social isolation can affect mental health and, conversely, it can be dependent on it. First, it is appropriate to address E. Ringel’s “pre-suicide syndrome,” which situates social isolation within the chronology of a suicidal crisis. Currently, social isolation is considered an integral part of suicide crisis assessment and a red flag indicating potential suicide with a risk of imminent action [55]. Among psychiatric disorders, the literature reviewed suggests that depressive disorders are closely linked to social isolation and suicide, with evidence of a bidirectional relationship between clinical depression and social isolation. Moreover, in several articles analyzed, the results reported were adjusted for depression to account for its confounding potential [15, 18, 21, 23, 26, 44, 45, 47]. Results diverge regarding whether social isolation should be considered as a suicidal risk factor independently from depression [15, 18, 23, 26, 33, 44, 51], leading to the assumption that social and mental health issues should be regarded as synergetic factors to improve the reliability of suicidality assessments. This view is supported by the mediation effects of both social isolation and depressive disorders on suicidality reported in some articles [37, 39–41].

In addition, social isolation is integral to the symptomatology of some mental disorders. This is the case for autism spectrum disorder and schizophrenia, for example. In both conditions, social functioning is generally impaired and interpersonal relationships are often poor [56], requiring appropriate consideration regarding their risk of suicide. One final clinical picture that should be considered is the hikikomori, initially described in Japan. Hikikomori refers to people who avoid social participation and relationships with people outside their family circle by confining themselves to their home or a room in their home for at least 6 months [48]. Hikikomori is a mental health condition involving both relational difficulties and a significant risk of suicide, potentially representing a theoretical model of social isolation in which suicide causality can be explored.

Psychopathology is a key element to account for when studying the link between social isolation and suicidality, hence the importance of considering the bidirectional relationship between social isolation and mental health issues.

#### Specific circumstances

Recent research has identified specific circumstances as a moderating factor in the relationship between social isolation and suicide. These circumstances should therefore draw specific attention to suicide prevention. In sexual minorities such as the Lesbian, Gay, and Bisexual (LGB) community, and ethnic minorities such as the Alaskan Native community, social isolation seems to have a different and greater impact on suicidality than it does in the general population [13, 42, 50]. Moreover, as a determinant of interpersonal relationships, culture should be considered when exploring the link between social isolation and suicide. Social isolation has probably a more substantial influence on suicidality in individualistic cultures than in collectivistic cultures such as the Chinese and Hispanic communities [7, 52]. Regarding the occupational sphere, some occupations and professional environments can facilitate social isolation, thus could lead to higher risks of suicidality [34, 40]. Unemployment, a known suicidal risk factor, can also reduce or even eliminate important social ties [14]. Finally, other forms of isolation must be taken into consideration. The physical isolation of detained individuals and the geographical isolation of people living in rural areas are two situations known to be associated with suicide [9, 14, 33, 39].

### Perspectives

#### Prevention targets

The possible causal relationship between social isolation and suicide and the opposite potential protective effect of social support have key implications for the fight against suicide. Associations and correlations have been identified in this literature review, including relationships between other variables and suicidality appearing to be mediated by social isolation and related social concepts [12, 13, 40, 50]. Consequently, social isolation might be a suicide risk factor that multiple actions should target to counteract the influence of individual risk factors. While there is a substantial body of literature suggesting social isolation as a major suicide risk factor, the response in terms of specific interventions is not commensurate with this proposal. This review has identified several variables that help clarify the link between social isolation and suicide, including age, gender, psychopathology, and other specific circumstances. Building on this information, groups at risk for the adverse consequences of social isolation should be routinely identified among the general population to benefit from preventive interventions, including targeted information campaigns in select environments such as schools for young people [47]. Such universal prevention initiatives could be complemented by raising awareness among healthcare providers and frontline social workers about the relationship between social isolation and suicide. Isolated persons can be identified by asking simple questions focusing on the individual’s degree of social isolation [23]. Tools such as the INQ can also be used to assess the level of thwarted belongingness experienced by individuals in the community [23].

#### Outreach

In addition to prevention strategies leveraging mental health services, proximity interventions that strengthen or create social ties, ranging from the family to the community environment, could be supported. Regardless of age, the focus should be placed on family interpersonal relationships [11, 24, 26, 27, 37]. Moreover, educational and work environments likely require specific interventions. Since school connectedness appears to be a strong protective factor for adolescents [9, 27, 41, 47], facilitating engagement in extracurricular activities, improving youth-adult networks, and strengthening the positive influence of youth with strong coping mechanisms toward their suicidal peers within school settings could help to reduce youth suicidality [9, 47]. For adults, fostering a strong sense of belonging in workplace environments and providing intensive support in situations in which the sense of belonging is threatened, especially in certain occupations facilitating isolation or during unemployment, seems to be key actions against the emergence of suicidality [14, 34, 40]. Finally, community integration through membership in organizations, among other things, looks crucial in the fight against suicide [10, 11]. Formal social relationships providing alternative sources of social support should be developed within society when required, for example, through home-based interventions with isolated older adults [19, 26].

Regarding specialized psychiatric care, insofar as psychiatric drug therapy does not involve social participation [48], socially involved and action-oriented psychotherapeutic approaches should be provided. More specifically, assumed influences of social isolation and social support on suicide suggest that family and group-based therapies should be favored over exclusively individual therapies [43]. From the perspective of the interpersonal theory of suicide, cognitive-behavioral therapeutic interventions targeting cognitive distortions of thwarted belongingness as well as participation in social activities can be considered [23]. The relationship with the therapist can also enhance the sense of belonging [18].

## Discussion

### General

The purpose of this review was to explore the relationship between suicide and a social variable, social isolation, as reported in the international literature. Our findings, drawn from theoretical models and a large number of empirical studies, suggest a basic relationship between suicide and social isolation and portray variations within this relationship related to age, gender, psychopathology, and specific circumstances. Furthermore, this review highlighted the potential protective effect of social support on suicide. Nevertheless, some social ties may be harmful rather than protective and promote suicidality, such as when the other person is suicidal or has ended their life or when someone is a victim of harassment.

The studies included in this review used a diversity of variables and questionnaires covering all kinds of areas of life to assess social isolation. Social isolation thus appears to be a general, overarching condition that arises from multiple social parameters [34]. As a result, in the context of suicidality assessments, social isolation must be viewed as a complex phenomenon with no set definition. This literature review focuses on social isolation as the objective portrayal of a social situation characterized by poor social ties. However, socially isolated individuals may not feel lonely, and vice versa [14, 35]. The concept of loneliness refers to the subjective aspect of the social condition of isolation. Loneliness can be described as a complex set of feelings encompassing reactions to the absence of intimate and social needs [57]. Consequently, a combined assessment of social isolation and loneliness may clarify a lived situation of isolation in relation to suicidality.

### Review strengths and limitations

The main strength of this literature review lies in the broad scope and varied nature of the articles examined. However, there are limitations pertaining to the methodological heterogeneity of the selected articles, complexifying comparisons and therefore limiting the repeatability of our analysis. First, most of the studies included were cross-sectional in design, although some used data extracted from longitudinal research [19, 20, 30, 33, 41, 50]. Few of the studies reviewed were longitudinal [15, 36, 38, 44], precluding the establishment of causality in the results and only allowing for correlations to be highlighted. In order to improve our understanding of the relationship between social isolation and suicide and to develop optimal suicide prevention by acting on the social environment, it is necessary to conduct longitudinal studies in the future [30, 47], in particular research evaluating the effectiveness of interventions aimed at supporting social connectedness.

Second, analysis procedures also varied from one study to another, particularly regarding whether confounding factors were taken into account. In the studies that controlled for confounding factors, the factors considered (e.g., age, sex, psychiatric disorder, socioeconomic status) were not always the same. The populations studied were also often different: samples or various sizes, more or less representative of the general population, of different age groups, gender, countries, ethnic origins, and cultures.

Third, the studies relied on many different measures of suicidality and social isolation or support, mainly self-reports [11, 13–17, 20, 22, 25–28, 30, 37, 39, 41, 43, 45, 46, 50, 51]. Variables representing social isolation varied and were assessed in several different ways. Several of the studies used the INQ [7, 21, 38, 40, 51], providing relative objectivity in measurement and improving the reliability of their results. However, in most studies, social isolation was determined based on variables selected as best approximating this concept, such as social network, single relationship status, or living alone. In addition, one study elicited the feeling of social isolation in an experimental manner (i.e., recalling a past experience, imagining a future experience, and inducing a sense of being ostracized at the time of the study), allowing for greater reproducibility and assessment reliability [15]. Suicidality was also measured in multiple ways, focusing on all stages of the suicidal process or, more globally, on the suicide risk. Less than half of the studies selected used the number of deaths by suicide as a variable [11, 14, 16, 17, 24, 25, 29, 30, 32, 36, 39, 42–45, 49]. The number of suicides is the most factual variable, but the rarity of this event often makes it necessary to assess suicidality through alternative measures. The multiplicity of methods used to assess social isolation and suicidality raises the need for harmonizing measures and even the development of a standardized measure for each of these variables in international research.

### Actuality and media consumption

There is a further risk factor of suicide, which is distally associated with social isolation: media consumption. At the beginning of the current health context of the pandemic in Covid-19, physical distancing was adopted as a primary strategy. Thus, there was an increasing reliance on social-platform outlets to connect to others [58]. Media content may be associated with increases in suicides. For example, the reporting of celebrity suicides appears to have had a notable impact on the total number of suicides in the general population [59]. Yet, the media can also have positive effects on suicidality. Indeed, media narratives of hope and recovery from suicidal crises appear to have a beneficial effect on suicidal ideation in individuals with some vulnerability [60].

## Conclusion

This literature review emphasizes a correlation between suicide and social isolation while suggesting that, conversely, social support has likely a protective factor against suicidality. This research also highlighted age, gender, psychopathology, and specific circumstances as essential variables that must be considered when studying the relationship between suicide and social isolation and developing intervention strategies targeting at-risk populations. Social isolation is a complex phenomenon that must be recognized as a major public health issue, a societal problem against which actions can be taken. However, there is a gap between the abundant literature showing the potential suicide risk associated with social isolation and the response in terms of appropriate interventions. Interventions must address social isolation in an interdisciplinary manner, through joint action involving the health and social sectors, on several levels ranging from prevention to therapy, and from local-scale proximity interventions to large-scale international policies.

This research received no specific grant from any funding agency, commercial or not-for-profit sectors.

## Data Availability

The data that support the findings of this study are available from two databases Medline via PubMeb and PsycINFO. Possible restrictions apply to the availability of these data.
